# Enhancing Pediatric Dental Care: The Influence of Virtual Reality

**DOI:** 10.1055/s-0044-1782193

**Published:** 2024-05-14

**Authors:** Sara Faisal Hamdy, Mohamed Sherif Mohamed Salah Farag, Yousra Samir Helmy, Asmaa Ali Abo-Elsoud

**Affiliations:** 1 Department of Pediatric and Preventive Dentistry and Dental Public Health, Faculty of Dentistry, Suez Canal University, Ismaillia, Egypt

**Keywords:** virtual reality, dental anesthesia, pain and anxiety

## Abstract

**Objective** The purpose of this study was to assess the effects of virtual reality (VR) in reducing pain and anxiety levels in children. The study also compared active and passive distraction methods using VR during the delivery of inferior alveolar nerve blocks (IANBs) in dental procedures in children.

**Material and Methods** The study comprised 45 preschool patients, aged between 4 and 6 years, with no prior dental anesthetic experience. The participants were randomly assigned to three groups based on the sort of management style: Group A used the tell-show-do technique, Group B engaged in passive distraction by watching cartoons using a VR headset, and Group C participated in active distraction by playing games using a controller with the VR headset. Pain and anxiety were evaluated using physiological measurements, namely by analyzing the variations in blood pressure, heart rate, and oxygen saturation before and after the administration of IANB. Psychological assessments were conducted using the Wong–Baker faces scale, Modified Dental Anxiety scale questionnaires, and Revised Face, Legs, Activity, Cry and Consolability scale after administering IANB.

**Results** The physiological outcomes revealed no statistically significant differences in blood pressure and oxygen saturation. However, there was a statistically significant increase in the heart rate in group A compared with groups B and C. In terms of psychological measurements, groups B and C exhibited a significant improvement in pain experience and a decrease in anxiety.

**Conclusion** This study concluded that VR reduced pain and anxiety levels in its passive and active forms.

## Introduction

Dental anxiety is a significant issue that affects children worldwide, with an estimated overall prevalence of roughly 23.9% according to a comprehensive review conducted in 2020. 1 2

Children who have high levels of dental anxiety tend to have worse oral health outcomes, including a higher occurrence of dental caries, and they also experience more pain and discomfort than nondentally anxious children. 2 3 Furthermore, it may exert detrimental effects on children's capacity to interact with others, thereby compromising their self-confidence and overall well-being. 4 Thus, the goal of pediatric dentists is to provide dental treatment in a calm, comfortable environment with minimum painful stimuli, consequently, reducing anxiety and fear and preventing the development of future dental phobia. 5

Promoting the practice of dentistry without causing pain is essential for diminishing fear and anxiety, allowing the provision of treatment, fostering a trusting connection between the dentist and patient, and promoting acceptance of future treatment. Administering local anesthesia is a crucial component, although, regrettably, it remains considered to be among the most difficult parts of pediatric dentistry. 6 The utilization of needles for local anesthetic is a prevalent factor causing dental fear, 7 inferior alveolar nerve blocks (IANBs) anesthesia is a commonly used technique in children, and due to its degree of difficulty, the infiltration technique may have a greater disturbing effect on children's conduct in the dental clinic. 7

Various psychological and pharmacological methods can be employed to alleviate patients' pain and anxiety while undergoing dental operations. Two often employed psychological strategies are tell-show-do and distraction techniques. 8 The tell-show-do technique is a method that involves providing verbal explanations of procedures using language that is suitable for the patient's level of development (tell). It also includes demonstrating the visual, auditory, olfactory, and tactile aspects of the procedure to the patient in a controlled and safe environment (show). Finally, the procedure is performed (do). 8

Distraction is a simply nonpharmacological pain management method that can be employed alongside conventional pain drugs to effectively regulate acute discomfort during medical operations. As per the Attention Pain Theory proposed by Eccleston and Crombez, 9 distraction can diminish the patient's available attentional resources for processing pain signals from neural receptors, leading to a decrease in the perceived intensity of pain. There are different types of distractions, distraction using gate way theory of pain, short time distractions, as chatting, and longtime distractions which could be audio (i.e., music) or visual (i.e., watching television screens on a silent mood) or both audio–visual distractions. 10 Audio–visual distraction can manifest in two ways: passively, by diverting attention through the senses of hearing and seeing, or actively, by engaging in a game. Nevertheless, the efficacy of conventional diversions in mitigating pain and fear is frequently constrained. 11 12

Virtual reality (VR) analgesia is an innovative and efficient pain distraction approach that shows great promise in alleviating suffering and enhancing the enjoyment of children during difficult medical procedures. 13 14 15 By combining hearing, seeing, and touching, VR engages multiple sensory modalities, offering a more immersive and interactive experience. 8

The essence of immersive VR is the user's illusion of being immersed inside the 3D computer-generated world. Patients wear a head-mounted display that blocks the patients' view of the real world, substituting it by computer-generated visual images and sound effects.

A growing number of studies have shown the effectiveness of VR for reducing pain. A study performed by Atzori et al. 15 supports the feasibility of VR as a distraction technique for pain management in children and adolescents and this study concluded that this psychological technique can help reduce pain during tooth extraction and dental fillings without side effects, and made dental procedures more fun.

A comprehensive analysis conducted by Padilha et al 16 affirmed that VR stands out as a highly efficacious approach to behavior management in pediatric dentistry. The review determined that VR not only effectively mitigates pain and anxiety in children undergoing dental procedures but also surpasses the effectiveness of traditional tools in achieving this outcome. Through its ability to provide an engaging and immersive experience, VR adeptly redirects the focus of young patients away from the clinical setting, thereby promoting a positive and enjoyable treatment experience.

The recent widespread production of immersive VR goggles has significantly enhanced the accessibility and affordability of VR headsets. This development coupled with the growing interest in nonpharmacological methods for pain management, along with children's increasing inclination toward technology and electronic games, whether on mobile phones, computers, or gaming consoles, positions VR distraction techniques as a promising avenue for future research. However, the effectiveness of highly immersive and active VR in reducing pediatric dental pain and alleviating dental anxiety during procedures remains uncertain. 16 Researchers, such as Snoswell and Snoswell, 17 have demonstrated the promising potential of VR in health care, particularly in the medical field, where it has shown substantial efficacy in supporting medical treatment efforts. Moreover, in dental education, VR is gaining recognition as a valuable tool for training dental students, with its utilization by dental schools witnessing a global rise, as noted by Moussa et al. 18

Hence, the objective of this investigation was to evaluate the efficacy of VR as a distraction method and to make a comparison between its passive and active iterations in contrast to the conventional tell–show–do technique strategies. This evaluation specifically focused on the context of IANB administration in preschool-aged children.

## Null Hypothesis

No discernible distinction in pain and anxiety levels was observed between the application and nonapplication of audio–visual distraction techniques employing VR glasses during the administration of IANB in preschool-aged children.

## Materials and Methods

Sample size calculation was performed using G*Power version 3.1.9.2, Faul et al, University Kiel, Germany. Copyright (c) 1992–2014. 19

The effect size d was 0.62 according to Felemban et al, 20 (85.48 ± 9.98) using alpha (α) level of 0.05 and beta (β) level of 0.05, that is, power = 95%; the estimated sample size ( *n* ) should be 45 samples (15 samples for each group).

The participants were recruited from the Outpatient Clinic of Pediatric Dentistry and Dental Public Health Department at the Faculty of Dentistry, Suez Canal University. Approval for the study was granted by the Research Ethics Committee (REC) at the Faculty of Dentistry, Suez Canal University (No.144/2018), adhering to the World Medical Association Declaration of Helsinki (version 2008). Written informed consent, signifying the legal guardians' agreement for their children to participate in the study, was obtained. The study procedures were thoroughly explained to both the patients and their parents.

### The Inclusion Criteria

The chosen participants were in the age range of 4 to 6 years, falling within the 3rd and 4th categories of the Frankel scale, 21 and exhibited overall good health. Inclusion criteria comprised those requiring dental intervention specifically for their lower primary second molars, who had not undergone anesthesia previously, and demonstrated a willingness to wear VR glasses while expressing an interest in watching cartoons or playing video games. 6 Exclusion criteria encompassed children with visual or auditory impairments, language barriers, or any history of prior invasive medical or dental trauma. 22

### Patients Grouping and Randomization

**Fig. 1:**
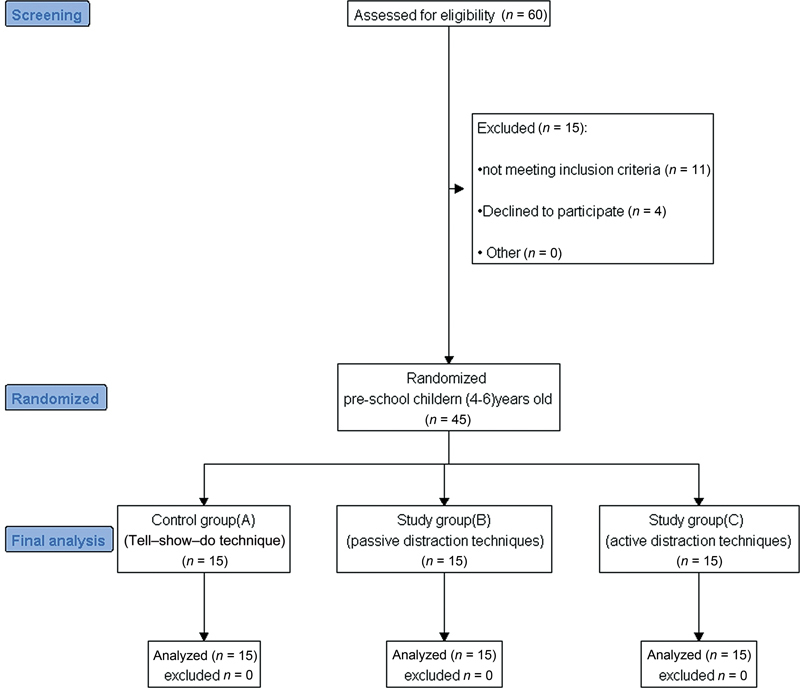
CONSORT flow diagram of the study.

This study consisted of 45 preschool patients, divided randomly into three equal groups, each 15 patients, based on the type of distraction technique used. Group A (control group) managed with tell–show–do technique, group B (Passive distraction group) managed with VR glasses where children watched cartoon series, and group C (Active distraction group) managed with VR glasses where children played video games. Randomization was performed by allowing each patient to select one card from a bowl containing 45 cards equally distributed between groups A, B, or C ( Fig. 1 ).

The VR goggles used was Samsung gear VR (Model-SM-R325NZVAXAR) headgear with S9 Samsung Mobile phone device, which acts as the headset's display and processor, the controller of the VR headset acts as a remote control to navigate the VR with ease or use as a gamepad.

“Baby shark VR dancing” show was chosen, its singing part was played as a passive distraction technique and its game part was played as an active distraction technique.

### Clinical Steps

**Fig. 2:**
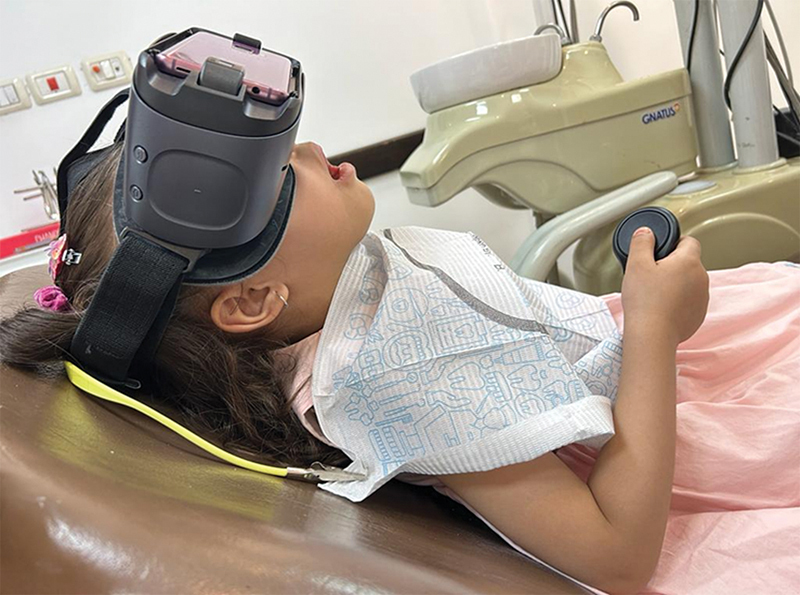
A 4.5 years old girl wearing VR head set ( **a** ) and playing video games by using the VR controller ( **b** ).

Instructions on the usage of VR goggles and controllers were provided to the patients in the study groups. They were given the opportunity to familiarize themselves with the VR device through a trial period of approximately 5 minutes before the commencement of dental treatment. Subsequently, the mobile device was switched to the flight mode, 23 as illustrated in Fig. 2 .

For the control group, the operator employed a friendly tone to explain to each child that a “magic water” would be used to make the tooth numb. The syringe, referred to as the instrument delivering the “magic water,” was presented along with the insertion of an anesthetic cartridge and a needle, the cover of which was removed during the administration of anesthesia. The child was then requested to open their mouth widely, close their eyes to shield them from the dental unit light, and raise their left hand if any discomfort was felt during the anesthesia procedure. The syringe was placed inside the child's mouth to simulate a block mandibular anesthesia, and the administration of the IANB was performed. 22

Following the drying of the injection area, a 20% benzocaine-flavored anesthetic topical gel was applied for 30 seconds. 22 The IANB was then performed using a 30-gauge needle and 1.8 mL of lidocaine 2% with 1:100,000 epinephrine local anesthesia. 22 The traditional nerve block anesthesia was performed with a long needle (35 mm lengths), (27 gauge), and 1.5 mL of lidocaine 2% with 1:100,000 epinephrine local anesthesia. The injection site, situated approximately 2 to 3 mm below the occlusal plane, was adjusted for preschool children at a lower level than in adults. 24 The operator palpated the coronoid notch with the thumb, pulled the buccal soft tissue laterally for visibility, and made the tissue taut. The needle, with the bevel upwards, was then slowly advanced until bony resistance was felt, followed by aspiration. Subsequently, 1.5 mL of the anesthetic solution was slowly deposited, taking an average of 1 minute and 15 seconds. 25 The entire anesthesia and dental treatment procedures were performed by a single operator for all patients.

The assessment methods included both physiological and psychological parameters.

#### Physiological Parameters

Blood pressure, pulse rate, and oxygen saturation were recorded directly by using the automated blood pressure device and pulse oximeter with small size calf for children, while the patient was sitting comfortably on the dental chair. 26

These physiologic measurements were recorded twice, before and immediately after the administration of IANB anesthesia, which took about average 1 minute and 10 seconds for each record. 25

#### Psychological Parameters

These parameters were recorded once after the administration of IANB anesthesia.

(1) Wong–Baker scale 27 : the patient was asked to point out the face that indicated the pain level they experienced on one of the six faces shown on the chart given to him. 23
(2) Modified Dental Anxiety scale 27 : were evaluated by asking patients five questions (multiple choice questions) to define their anxiety level toward the next dental procedures on the dental chair 22
–Both assessments were done by one operator and under supervision, which took about average of 1 minute. 28
(3) The Legs, Activity, Cry, Revised Face and Consolability (R-FLACC) Scale 29 : the anesthetic procedure was video recorded so all the body responses of the patient were then evaluated by the supervisor to determine the children's behavioral score,

All data were gathered, computed, organized, and subjected to statistical analysis through the application of specific statistical tests. To assess the normal distribution of the sample, a normality test (Kolmogorov–Smirnov) was conducted.

## Results

Descriptive statistics, presented as mean ± standard deviation (SD), were computed for the data analysis. The Kruskal–Wallis test was employed to assess differences between groups for each variable, while the chi-square test was utilized to evaluate qualitative data distinctions among the groups. A paired sample *t* -test was conducted to compare the two groups. A significance level of *p* ≤ 0.05 was considered statistically significant. All statistical analyses were performed using the SPSS software for Windows version 22.0 (Statistical Package for Social Science, Armonk, NY: IBM Corp). The chosen significance level for all analyses was set at 0.05 ( *p* -value ≤ 0.05).

### Regarding Gender

There was no significant difference in gender between all the groups ( Table 1 ).

**Table 1:** Gender

	Gender	Control (A)	Group (B)	Group (C)	Chi-square	*p* -Value 0.05
4–6 y	M	7	6	8	0.5357	0.765 ns
	F	8	9	7		

Abbreviation: ns, nonsignificant.

### Regarding the Physiological Parameters

(1) Blood pressure (BP): The results, as indicated by a dependent *t* -test, revealed no statistically significant difference between preanesthesia and postanesthesia measurements in both systolic blood pressure (SBP) and diastolic blood pressure (DBP) across all groups ( Table 2 ).
(2) Heart rate: The findings, analyzed through a dependent *t* -test, demonstrated a noteworthy elevation in the heart rate solely within group A when comparing measurements before and after anesthesia ( Table 2 ).
(3) Oxygen saturation: The results, assessed through a dependent *t* -test, indicated an insignificantly different change in oxygen saturation levels before and after anesthesia across all groups ( Table 2 ).

**Table 2:** Physiological parameters results

**(1) Blood pressure**									
**Change in SBP**						**Change in DBP**			
		**Mean**	**SD**	**T**	**Sig**	**Mean**	**SD**	**T**	**Sig**
Group A	Before	105.60	17.18	0.481	0.638 ns	70.67	9.29	−0.353	0.729 ns
	After	104.00	12.47			71.73	11.42		
Group B	Before	105.00	10.67	−1.45	0.169 ns	70	6.8	−1.682	0.115 ns
	After	107.73	9.78			73	6.22		
Group C	Before	100.47	9.11	−1.32	0.209 ns	66.87	10.46	−1.972	0.069 ns
	After	103.00	9.38			70.93	7.5		
**(2) Heart rate**						**(3) Oxygen saturation**			
Group A	Before	95.87	15.22	−3.9	0.002 a	96.73	1.49	−0.878	0.395 ns
	After	109.27	14.05			97.13	1.64		
Group B	Before	97.46	13.59	−1.69	0.114 ns	98	0.85	−0.269	0.792 ns
	After	101.67	17.04			98.07	0.7		
Group C	Before	102.06	12.63	−1.93	0.07 ns	97.6	0.99	−0.77	0. 20 ns
	After	106.87	15.29			98.13	0.64		

Abbreviations: DBP, diastolic blood pressure; ns, nonsignificant; SBP, systolic blood pressure; SD, standard deviation.

a Significant.

### Regarding the Psychological Parameters

(1) Wong–Baker Faces Pain Rating Scale: The results using the Kruskal–Wallis test at *p* < 0.05 showed a highly significant increase in group A (the heights mean 4.8 ± 2.366) in comparison to B and C groups ( Table 3 ).
(2) Modified Dental Anxiety scale: The results using the Kruskal–Wallis test at *p* < 0.05 showed a highly significant increase in group A (the heights overall mean 2.97 ± 1.11) in comparison to B and C groups ( Table 3 ).
(3) R-FLACC scale test changes: The results used the Kruskal–Wallis *t* -test showed highly significant differences between all groups for R-FLACC scores at *p* < 0.05. A group gave the highest mean scores for R-FLACC scores in comparison to group B and C ( Table 4 ).

**Table 3:** Psychological parameters results

		Groups	Mean	Standard deviation	Minimum	Maximum	Mean rank	Kruskal–Wallis	Significance
Changes in 1-Wong–Baker Faces Pain rating Scale		A	4.8	2.366	2	10	35.7	23.37	<0.01 **
		B	0.933	1.28	0	4	16.87		
		C	1.2	2.597	0	10	16.43		
Changes in 2-Modified Dental Anxiety Scale	Q1	A	2.73	0.88	1	4	36.8	34.54	<0.01 **
		B	1.07	0.26	1	2	16.7		
		C	1	0	1	1	15.5		
	Q2	A	2.73	1.28	1	5	33.83	26.53	<0.01 **
		B	1.07	0.26	1	2	18.17		
		C	1	0	1	1	17		
	Q3	A	3.33	0.98	1	5	36.6	28.39	<0.01 **
		B	1.2	0.41	1	2	16.2		
		C	1.2	0.41	1	2	16.2		
	Q4	A	2.33	0.9	1	4	33.67	20.2	<0.01 **
		B	1.33	0.49	1	2	20.83		
		C	1	0	1	1	14.5		
	Q5	A	3.73	1.03	2	5	37.6	32.62	<0.01 **
		B	1.2	0.56	1	3	16.37		
		C	1.07	0.26	1	2	15.03		
	Over all	A	2.97	1.11	1	5	34.8	22.19	<0.01 **
		B	1.17	0.415	1	3	17.4		
		C	1.05	0.226	1	2	15.04		

* Mean significant *p* -value ≤ 0.05. **Highly significant *p* -value ≤ 0.01.

**Table 4:** R-FLACC scale test changes

		Mean	Standard Deviation	Minimum	Maximum	Mean Rank	Kruskal–Wallis	Significance
Face	A	1	0.53	0	2	38.0	42.18	<0.001 **
	B	0.2	0.41	0	1	15.5		
	C	0.07	0.26	0	1	15.5		
Legs	A	0.87	0.52	0	2	35.67	23.15	<0.001 **
	B	0.47	0.52	0	1	14.33		
	C	0.47	0.52	0	1	19.00		
Activities	A	1.00	0.38	0	2	36.73	35.61	<0.001 **
	B	0.27	0.46	0	1	15.00		
	C	0.07	0.26	0	1	17.27		
Cry	A	0.87	0.74	0	2	37.33	34.28	<0.001 **
	B	0.47	0.64	0	2	14.5		
	C	0.33	0.49	0	1	17.17		
Consolability	A	0.87	0.52	0	2	37.33	35.49	<0.001 **
	B	0.27	0.46	0	1	14.5		
	C	0.07	0.26	0	1	17.17		

Abbreviation: R-FLACC, Legs, Activity, Cry, Revised Face and Consolability.

* Mean significant *p* -value ≤ 0.05; **Highly significant *p* -value ≤ 0.01.

## Discussion

VR, in both its passive and active modalities, effectively mitigated pain and anxiety levels during the administration of IANB local anesthesia in preschool children. This investigation specifically focused on assessing pain and anxiety during the delivery of local anesthesia, which constitutes the most discomforting and stressful aspect of dental treatment. Unfortunately, it remains an important barrier for many children to receive proper dental treatment. 30 The IANB was used, in this study, as it is the most profound dental anesthesia, especially in treating inflamed pulp, but it is considered as a painful anesthetic technique when compared with buccal infiltration. 22 31 32

This study included preschool children, aged 4 to 6 years, as they are the most difficult to treat and usually show more disruptive behavior. 33 Patients, who had never received dental anesthesia, were chosen to avoid any previous psychological dental trauma. Cooperative and relaxed patients were randomly selected for reliable pain and anxiety test results. Pulse oximeter needed steady, unmoving hands to give a correct value. 34 Children included were medically free to avoid any adverse effects on the tested physiological measurements. Patients who had any visual or auditory defects, any previous bad experience, either dentally or medically, were excluded as distraction techniques are not effective in these individuals. 21

Physiological changes have shown to be reliable indicators of anxiety levels in patients before and after painful or traumatic procedures, such as dental anesthesia. 23 Therefore, in this study, blood pressure, pulse rate, and oxygen saturation were used in conjunction with other psychological parameters, as the psychological parameters alone could be misleading due to the potential limitations in children's cognitive and linguistic skills. 7

The operator evaluated the Modified Dental Anxiety test, and the supervisor, through video assessments, ensured objectivity to prevent bias toward a particular technique. Before assessing the child's dental anxiety, the operator underwent training and calibration. The supervisor re-evaluated videotaped dental sessions of three patients to assess the interexaminer reliability of the Wong–Baker Faces scale and Modified Dental Anxiety test using the Weighted Kappa test. The resulting values, ranging from 0.70 to 1.00, indicated a high level of reliability. 35

Concerning physiological outcomes, pulse rate and blood pressure served as parameters to gauge the effectiveness of behavior guidance techniques in alleviating dental fear and anxiety. In a study by Pande et al, 8 no significant difference in SBP and DBP was observed before and after the administration of IANB anesthesia across all groups. This finding aligns with the results of Al-khotani et al.'s study, 37 where no significant differences in SBP and DBP were noted before, during, and after anesthesia administration when comparing two groups (no distraction and VR group), albeit in a different age group (7–9 years). The lack of significant changes in blood pressure may be attributed to the relatively short duration of anesthesia administration, as blood pressure may require a longer time to manifest alterations compared with the brief injection period.

Conversely, the findings diverged from those of Singh et al, 36 who conducted a study involving children aged 6 to 12 years. In their investigation, a comparison between a control group and a music group utilizing solely audio distraction techniques revealed a significant decrease in SBP when compared with the baseline measurements.

However, there was a significant increase in heart rate, before and after anesthesia, in the control group, indicating that children were more susceptible to stress and anxiety during the administration of IANB. Studies conducted by Mitrakul et al, 33 Al-khotani et al, 37 and Buldur and Candan 38 on children found that using VR distraction during dental treatment led to reduced heart rates compared with treatment without VR. However, Al-Halabi et al 25 did not find a significant difference in heart rate while using VR glasses. A study by Attar and Baghdadi 39 showed that using an iPad for active distraction resulted in lower average heart rates during treatment in children aged 4 to 8-year-old compared with using VR eyeglasses for passive distraction.

Concerning the results of oxygen saturation, there was no significant difference before and after the administration of IANB in all groups, this may be due to the short time of administration of anesthesia, which did not give a chance for oxygen saturation to be changed. This is in accordance with Niharika et al 10 , who conducted a study on 4 to 8-year-old children and found that there were no significant changes in oxygen saturation in the same patient undergoing two sessions of dental treatment one using the VR distraction technique and one without.

Regarding psychological outcomes, notable differences were observed in overall disruptive behavior between the control group and the VR groups, with children in the VR groups exhibiting improved behavior and displaying a positive response. The Wong–Baker test results demonstrated a highly significant disparity between group A and groups B and C, as the majority of patients in group A chose the “no hurt” score. These findings align with studies by Aminabadi et al 40 and Niharika et al 10 . However, a study conducted by Al-Halabi et al 25 reported no significant difference in the Wong–Baker test results when comparing VR distraction technique, tablets, and control groups. This discrepancy could be attributed to the possibility that the use of VR eyeglasses does not offer additional advantages in managing child behavior and alleviating pain in older children (6–10 years) during IANB administration.

Furthermore, a highly significant difference was also observed, when comparing the control group with VR groups, using the Modified Dental Anxiety tests, as most of the patients in the VR groups selected “not anxious.” This result is in agreement with Aminabadi et al. 40 and Niharika et al. 10

Finally, the distracting effects of VR were found to reduce children's physical distress in the VR groups, as demonstrated by significant differences in the R-FLACC test. A study by Mitrakul et al 18 showed that VR eyeglasses effectively reduced child's physical distress preoperatively and the first use of high-speed hand piece. However, R-FLACC score during the remaining restorative treatment was not significantly decreased. Furthermore, studies by Bagattoni et al 41 42 conducted on special care needs children, aged from 5 to 10 years, found significant differences with the R-FLACC scale in favor of the audiovisual distraction technique. On the contrary, Al-Halabi et al 25 did not find any significant differences in the FLACC scale between the VR distraction technique versus the tablet, and control groups.

Concerning active and passive distraction techniques, this study did not identify a significant difference between both approaches. However, a study by Attar and Baghdadi 39 suggested that active distraction, using a tablet, enhances the child's visual, mental, and motor participation, providing superior anxiolysis and analgesia compared with passive distraction and the reason could be the use of VR glasses in both active and passive distraction in this study.

Researchers have put forth explanations for the pain-reducing role of VR. 13 14 The immersive nature of VR captures the brain's attention, limiting its capacity to process incoming pain signals, as suggested by Fakhruddin et al. 43

Additionally, Felemban et al 20 proposed that VR glasses, by blocking vision, might induce a feeling of isolation from the real world and increase anxiety related to an unpleasant stimulus. Despite this, the patients, whose view was obstructed by the VR glasses, expressed a preference for dental treatment with their use. This preference may be attributed to the child's personal interest in technology.

The utilization of VR in managing dental anxiety operates through various mechanisms. Cognitive distraction is a primary mechanism, diverting attention from the dental procedure and reducing focus on negative thoughts and fears, ultimately alleviating anxiety. 18 Moreover, VR creates a relaxing and calming environment for the child during the dental visit.

Furthermore, the active form of VR empowers children by providing interactive elements and customizable experiences, allowing them to have a sense of control over their environment. This empowerment is particularly beneficial during dental visits, contributing to a positive and empowering experience.

## Conclusion

This investigation concluded that both passive and active forms of VR effectively lowered pain and anxiety levels, as evidenced by a significant reduction in heart rate during IANB delivery in preschool children. The Wong–Baker rating scale exhibited a noteworthy difference in both passive and active groups compared with the control group. Additionally, both the Modified Dental Anxiety scale and R-FLACC results demonstrated significant distinctions between the passive and active groups when compared with the control group. These findings underscore the considerable potential of VR as a valuable tool in the effective management of dental pain and anxiety.

## Recommendations

Ensuring the widespread adoption of VR technology in pediatric dental settings requires attention to both accessibility and affordability. It is crucial to develop standardized protocols and guidelines for the systematic integration of VR in the management of dental anxiety in children. Additionally, considering the synergy of VR with established behavior management techniques can enhance overall effectiveness. Further research is imperative to assess the long-term efficacy of VR interventions and their influence on children's oral health outcomes.

## Limitations

Incorporating VR into pediatric dental practice comes with challenges, including ethical considerations, technical limitations, individual differences, and associated costs. Additionally, some children may be hesitant to use VR glasses, as they may feel isolated from the real world, intensifying anxiety related to potential discomfort. It's important to note that preschool children may provide inaccurate responses when using self-reported scales. Tailoring VR content to suit the age-specific needs of each group is essential for effective implementation.
